# Cytotoxic effects of zinc oxide nanoparticles on cyanobacterium *Spirulina (Arthrospira) platensis*

**DOI:** 10.7717/peerj.4682

**Published:** 2018-06-01

**Authors:** Sinouvassane Djearamane, Yang Mooi Lim, Ling Shing Wong, Poh Foong Lee

**Affiliations:** 1 Department of Biomedical Science, Faculty of Science, Universiti Tunku Abdul Rahman, Kampar, Perak, Malaysia; 2 Department of Pre-Clinical Sciences, Faculty of Medicine and Health Sciences, Universiti Tunku Abdul Rahman, Sungai Long, Selangor, Malaysia; 3 Department of Biotechnology, Faculty of Health and Life Sciences, INTI International University, Nilai, Negeri Sembilan, Malaysia; 4 Department of Mechanical & Material Engineering, Lee Kong Chian Faculty of Engineering and Science, Universiti Tunku Abdul Rahman, Sungai Long, Selangor, Malaysia

**Keywords:** Zinc oxide nanoparticles, *Spirulina platensis*, Microalgae, Cytotoxicity

## Abstract

**Background:**

The extensive usage of zinc oxide nanoparticles (ZnO NPs) in industrial and consumer products raises the risk of releasing their residues into the aquatic environment. The presence of ZnO NPs in the aquatic environment could potentially cause cytotoxic effects on aquatic organisms. Thus, investigating the cytotoxic effects of ZnO NPs on microalgae, which form the base for the food web of aquatic biota, is essential to gain information regarding the ecotoxicological effects of metallic oxide nanoparticles in the aquatic ecosystem. Therefore, the present study has investigated in detail the assorted cytotoxic effects of ZnO NPs on *S. platensis* using various concentrations of ZnO NPs (10–200 mg/L) from 6 to 96 h to explore the dose- and time-dependent cytotoxic effects.

**Methods:**

The cytotoxic effects were all assessed through quantification of loss in cell viability, reduction in biomass and decrease in photosynthetic pigments such as chlorophyll-a, carotenoids and phycocyanin. The surface interactions of nanoparticles and the subsequent morphological alterations on algal cells were examined by optical and scanning electron microscopy (SEM). The intracellular alterations of algal cells were studied using transmission electron microscopy. Furthermore, Fourier transformed infrared (FTIR) spectrum was obtained to investigate the involvement of algal surface biomolecules in surface binding of ZnO NPs on algal cells.

**Results:**

The treatment of ZnO NPs on *S. platensis* exhibited a typical concentration- and time-dependent cytotoxicity. Results showed a significant (*p* < 0.05) cytotoxicity from 24 h onwards for all tested concentrations of ZnO NPs. The maximum cytotoxicity on algal cells was achieved at 96 h of exposure to ZnO NPs. In comparison with control, the algal cells that interacted with 200 mg/L of ZnO NPs for 96 h showed 87.3 ± 1% loss in cell viability, 76.1 ± 1.7% reduction in algal biomass, 92.5 ± 2.2%, 76.2 ± 2.2% and 74.1 ± 3.4% decrease in chlorophyll-a, carotenoids and phycocyanin contents respectively. Our study confirmed the cytotoxicity of ZnO NPs through the algal growth inhibition with 72 h EC_10_ and EC_50_ values of 1.29 and 31.56 mg/L, respectively. The microscopic examinations of the algal cells that interacted with ZnO NPs showed severe cell membrane and intracellular damage. The SEM EDX spectrum of ZnO NPs treated algal biomass evidenced the surface accumulation of zinc in the biomass. Finally, the FTIR spectrum confirmed the involvement of amino, hydroxyl and carboxylic groups of algal cell wall in the surface interaction of ZnO NPs on the algal cells.

**Discussion:**

The results showed that the treatment of ZnO NPs on *S. platensis* triggered substantial cytotoxicity and caused cell death. Hence, *S. platensis* could be potentially used as a bioindicator for testing toxicity of ZnO NPs in aquatic environment.

## Introduction

Nanotechnology development is eminent for industrial applications. The zinc oxide nanoparticle (ZnO NP) is one of the most widely used nanoparticles, and is being utilized in the production of pigments, semiconductors, UV protection films, chemical sensors, modern sunscreens and hair care products due to its adsorption ability, large surface area, transparency, UV absorption efficiency and chemical stability (Dastjerdi & Montazer, 2010; Osmond & Mccall, 2010; Gunawan et al., 2013). Furthermore, the antibacterial property of this particle has extended its application in the pharmaceutical and food industries (Prach, Stone & Proudfoot, 2013; Song et al., 2010).

The extensive application of ZnO NPs in consumer products certainly raises the imperative question of environmental safety. Upon release into the aquatic environment, these metallic oxide nanoparticles might carry potential adverse effects on the ecosystem and human health. Water pollution due to nanoparticles (NPs) occurs as a consequence of usage of NPs in consumer products as well as accidental spillages or permitted discharge of industrial effluents through sewage containing NPs in waterways and aquatic systems (Hardman, 2006; Thomas et al., 2011; Tsao, Chen & Wang, 2011). Nanoparticles transfer their hazard via aquatic environment (Bernhardt et al., 2010), and therefore, polluted aquatic systems create a huge adverse impact on biological ecosystems and bring harm to human health through direct exposure to NPs via skin contact, inhalation of polluted water aerosols, direct ingestion of contaminated drinking water or intake of vegetables and edible microalgae that adsorbed with NPs (Daughton, 2004; Kim, Hong & Ahn, 2013). Hence, the adverse effects of ZnO NPs evidently need to be assessed on various aquatic organisms as an integral part of environmental risk assessment (Bhuvaneshwari et al., 2015; Hazeem et al., 2016; Prach, Stone & Proudfoot, 2013).

Previous toxicity studies using metallic nanoparticles have evidenced that these particles are toxic to algae (Xu et al., 2013), bacteria (Li, Lin & Zhu, 2013), crustaceans (Blinova et al., 2010) and fish (Zhu et al., 2008). A large surface area of ZnO NPs endows them with high electron density and high reactivity to interact with biomolecules contributing to high bio-toxicity (Pisanic, Jin & Shubayev, 2009). Algae are largely dispersed in fresh water and they are the primary producers in the food chain; therefore, consequently, any disturbance in their growth reflects a disturbance in the food chain of aquatic system (Auffan et al., 2011). Alga is an organism that is sensitive to metallic contaminants when compared to fish and invertebrates (Zhou et al., 2014). Therefore, they can potentially be used as a biosensor to monitor water quality and aquatic toxicity (Franklin et al., 2007). Investigating the toxicity of ZnO NPs on alga is important to develop a strategy on assessing the potential adverse effects of engineered NPs in the aquatic environment (Maynard et al., 2006). Marine microalgae are good food source for marine organisms and play an important role as primary producers in the oceans. Thus, the assessment of NPs effects on marine microalgae is also an essential step to predict their adverse effects on marine food web and on the entire ecosystem they endure (Manzo et al., 2013).

*S. platensis* is a prokaryotic cyanobacterium, also a marine alga, that has been commercially produced for over 30 years in use of vitamin supplements, food dyes, aquaculture, pharmaceuticals and nutraceuticals (Abdulqader, Barsanti & Tredici, 2000). Prior studies have proven the capacity of *S. platensis* to accumulate the trace metals (Al-Dhabi, 2013; Dazhi et al., 2003). A study done by Lone et al. (2013) confirmed the toxic effects of ZnO NPs (10 mg/L) to *S. platensis* on day 10. Furthermore, authors have opined that NPs toxicity in the aquatic ecosystems due to various anthropogenic activities may cause harmful effects in its nutritional quality through biochemical and physiological alterations. Hence, further information concerning the toxicity effects of ZnO NPs on *S. platensis* in semi chronic exposure conditions may afford insights to develop methods to test the environmental contamination of ZnO NPs in nutrient microalgae. Therefore, *S. platensis* was selected as the model organism in this study to test the concentration- and time- dependent cytotoxicity effects of ZnO NPs, with the expectations that *S. platensis* could be a bioindicator for ZnO NPs toxicity in aquatic environment including marine ecosystem. Moreover, the study findings may be useful to identify the contamination of ZnO NPs during the industrial cultivation of nutrient microalgae.

In this study, the dose- and time-dependent cytotoxic effects of ZnO NPs on *S. platensis* were investigated through the determination of cell viability, biomass and photosynthetic pigments’ content. The surface interactions of NPs and the morphological alterations of algal cells were studied using phase contrast and scanning electron microscopy, while the intracellular alterations were confirmed by transmission electron microscopy. The FTIR and SEM EDX analyses were then used to confirm the involvement of algal cell wall in surface binding of NPs on algal cells and the accumulation of ZnO NPs in the algal biomass respectively.

## Materials & Methods

### Primary characterization of ZnO NPs

Zinc oxide nanoparticle (particle size >100 nm) powder was purchased from Sigma-Aldrich. The primary particle size was determined using scanning electron microscope (S-3400N, Scanning Electron Microscope; HITACHI, Tokyo, Japan), operated at an acceleration voltage of 20 kV. The X-ray energy dispersive spectroscopy (EDX) spectrum was obtained to confirm the chemical composition of ZnO NPs. The crystalline nature of the particles was assessed by X-ray diffractometer (Lab X, XRD-6000; Shimadzu Corp., Kyoto, Japan), operated at a voltage of 40 kW and a current of 30 mA with CuK *α* radiation *λ* = 1.5406 in the scan range of 2Θ = 10–80° to obtain the images.

### Cultivation of microalga

The cyanobcterium *Spirulina (Arthrospira) platensis* stock culture was purchased from UTEX1926 (University of Texas Culture Collection, Austin, TX, USA). *S. platensis* was cultivated in Spirulina medium with pH 9.0. This blue green microalga was maintained in Erlenmeyer flasks under 17–20 µmol photons/m^2^/s illumination using cool white fluorescent lamp from the height of 25 cm in 16:8 h light/dark regime at a temperature of 22 ± 1 °C.

### Exposure of microalga to NPs

**Figure 1:**
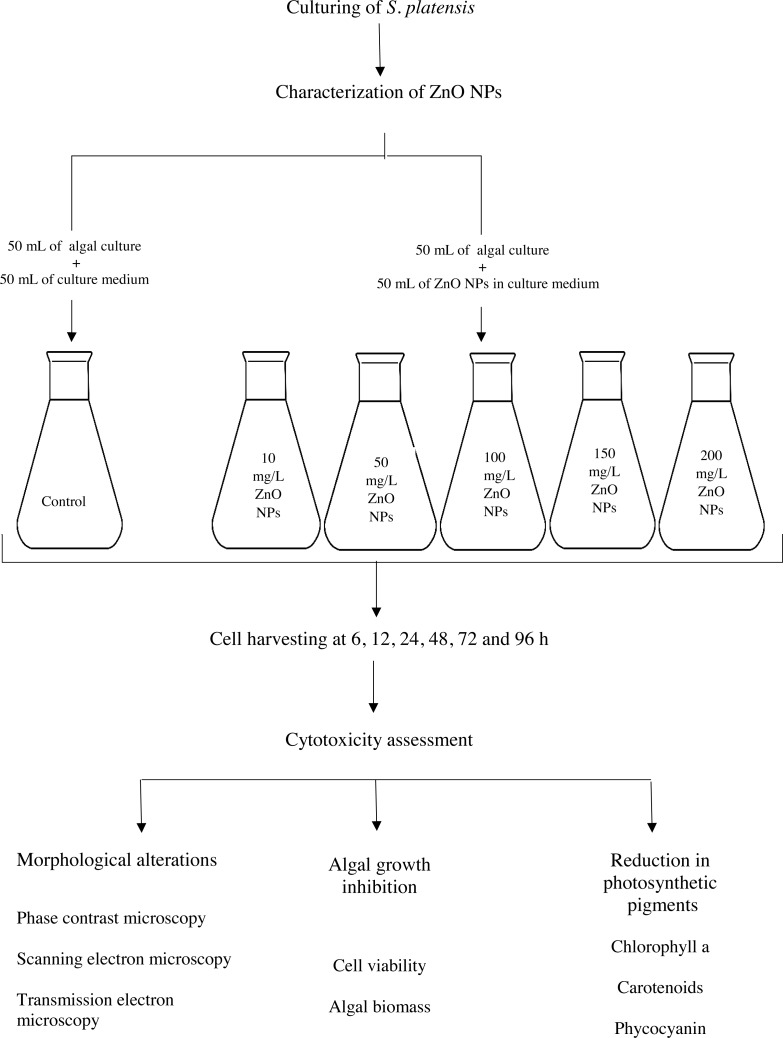
Experimental workflow of this study.

A stock solution (400 mg/L) of ZnO NPs was prepared by using the culture medium as a solvent and sonicated for 30 min at 40 kHz to prevent aggregation of NPs. The stock solution was diluted with culture medium to obtain different concentrations of ZnO NPs at 10, 50, 100, 150 and 200 mg/L respectively. The algal cells from day 5 of the culture, with an initial cell density of 1 × 10^5^ cells/mL, were exposed to 10, 50, 100, 150 and 200 mg/L of ZnO NPs in 250 ml Erlenmeyer flask for a period of 96 h along with the control that was devoid of NPs. The interacted algal cells together with control cells were subjected for cytotoxicity assessment at 6, 12, 24, 48, 72 and 96 h respectively as shown in Fig. 1.

### Cytotoxicity assessment

#### Determination of cell viability

The control and ZnO NPs interacted cells were loaded in Neubauer cell counting chamber (Marienfeld, Germany). The number of intact cells without any distortion in shape and size were counted as viable cells. The percentage of loss in cell viability of NPs interacted cells was calculated with respect to the control cells.

#### Quantification of biomass

The algal biomass was determined by using spectrophotometer (Genesys 20; Genesys, London, UK) at 560 nm with the culture medium as blank (Comotto et al., 2014). This investigation included an additional control particle-only control (cell-free) to serve as a reference to obtain the absorbance at 560 nm that corresponds to the algal biomass (Gunawan et al., 2013). The percentage of reduction in biomass was eventually determined with respect to the control.

#### Measurement of the photosynthetic pigments

To measure the photosynthetic pigments, 3 ml of treatment and control cell suspensions were centrifuged at 5,000 rpm for 10 min. The resulting pellet was washed twice in 1X phosphate buffer saline (PBS) to remove unbound particles and the pigments were extracted in 100% methanol at 65 °C for 60 min or until the cell debris were almost colorless (Tocquin, Fratamico & Franck, 2012). The extracted pigments in the supernatant were measured with the spectrophotometer (Genesys 20; Genesys, London, UK) at 470, 653 and 666 nm. Chlorophyll-a and carotenoids were quantified using the equations of Lichtenthaler & Wellburn (1985) (Deniz, Saygideger & Karaman, 2011). For phycocyanin estimation, glass pearl was added to the washed pellet and sonicated in the ultrasonic bath (40 kHz) for 60 min for extraction of pigment. The pigment containing supernatant was measured at 652 and 620 nm by spectrophotometer. The phycocyanin content (mg/mL) was calculated by using Eq. (1) (Moraes et al., 2011).

(1) \begin{eqnarray*}\text{Phycocyanin (mg/mL)}: \frac{{A}_{620}-0.474{A}_{652}}{534} \end{eqnarray*}

*A- absorbance at the specific wavelength.

### Surface interaction of NPs with algal cells

The optical and scanning electron microscopic studies were conducted to examine the surface interaction of NPs on algal cells and the subsequent morphological alterations of interacted cells. About 10 µl of algal suspension, after each interaction period, was loaded onto a glass slide and sealed with cover glass and observed under phase contrast microscope (Nikon Eclipse TS 100; Nikon, Tokyo, Japan). For SEM, about 5 ml cell suspension was centrifuged at 5,000 rpm for 10 min and the pellet was washed twice with 1X PBS to remove unbound NPs. The pellet was freeze-dried overnight to remove moisture (Dmytryk, Saeid & Chojnacka, 2014) and the freeze-dried algal cells were subjected for sputtered coating (Sputter Coater SC7620, HITACHI, Japan). The sputtered specimens were examined through SEM (S-3400N, Scanning Electron Microscope, HITACHI, Japan) with EDX analysis.

### Transmission electron microscopy (TEM)

Deformity in cellular organelles was assessed by transmission electron microscopy (TEM Libra 120; Zeiss, Oberkochen, Germany). The ultrathin sections of control cells and cells treated with 200 mg/L of ZnO NPs for 96 h were attached onto the copper grid and examined under transmission electron microscope for trans-sectional studies of algal cells.

### Fourier transformed infrared spectrometer analysis

Involvement of surface functional groups of algal cells in the surface interaction of ZnO NPs with the cells was analyzed using Fourier transformed infrared spectrometer Nicolet iS10 FT-IR spectrometer (Thermo Fisher Scientific, Waltham, MA, USA). A 5 ml of cell suspension was centrifuged for 10 min at 5,000 rpm. Then, the pellet was washed twice with 1X PBS and freeze-dried (Dalai et al., 2014). The dried algal cells were subjected for FTIR analysis and the infrared spectra were recorded at a resolution of 32 cm^−1^ over the range of 4000-501 cm^−1^.

### Statistical analysis

The experiments were carried out in triplicates and the results are presented as mean ± standard deviation. Shapiro–Wilk test was applied to test the normal distribution of data. Then, One-way analysis of variance (ANOVA) followed by Tukey’s post-hoc test for multiple comparisons (SPSS version 22; SPSS, Chicago, IL, USA) was used for analyzing the significant level of this empirical study. The level of significance was accepted at *p* value <0.05. The effective concentration (EC_10_ & EC_50_) to evaluate the toxic potential of ZnO NPs was calculated using the EPA Probit Analysis program (version 1.5; Enviromental Protection Agency, 2012) with 95% confidence interval.

## Results

### Characterization of ZnO NPs

**Figure 2:**
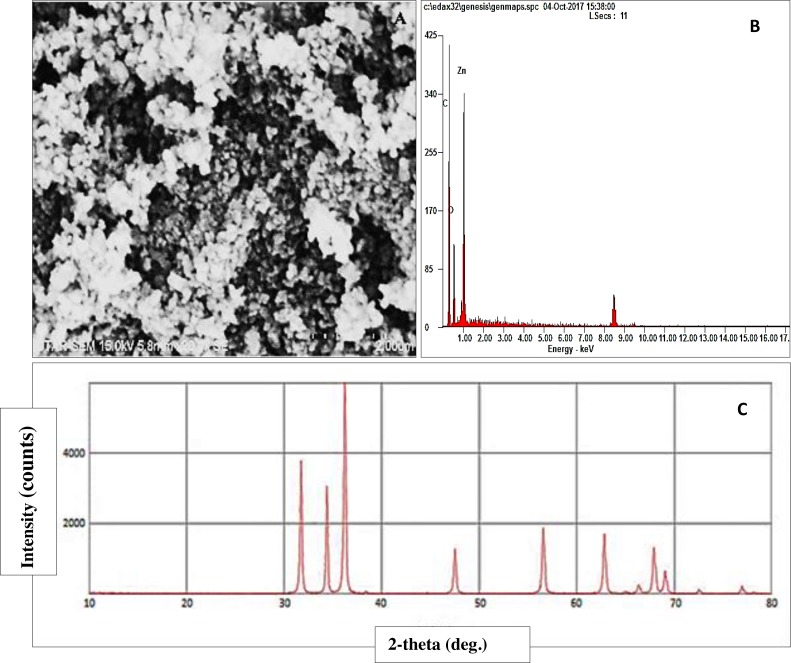
Characterization of ZnO NPs. SEM image (A), EDX image (B) and XRD pattern (C) of ZnO NPs.

SEM image of ZnO NPs is displayed in Fig. 2A. Spherical-shaped NPs were observed in the agglomerated state and the size of the NPs was determined to be around 44.6 nm with the size ranging from 39.7–49.6 nm. The EDX spectrum of ZnO NPs (Fig. 2B) confirmed the presence of zinc and oxygen in ZnO NPs powder. Figure 2C shows the XRD pattern of ZnO NPs that confirmed the hexagonal wurtzite crystalline structure of ZnO NPs.

### Cytotoxic assessment of ZnO NPs on *S. platensis*

#### Loss of cell viability

**Figure 3:**
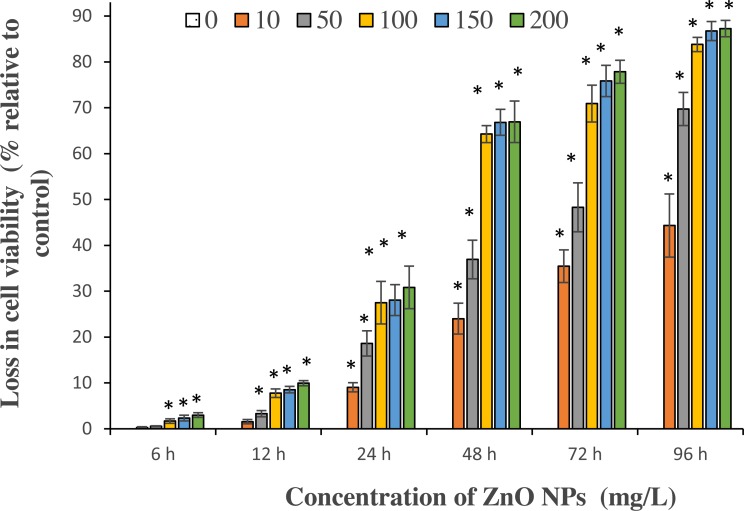
Loss in cell viability. Percentage of loss in cell viability against different concentrations of ZnO NPs for the exposure duration from 6 to 96 h. *, significant difference at *p* < 0.05 between the control and the tested concentrations at the specific time period.

A significant (*p* < 0.05) loss in viable cell counts of *S. platensis* was observed among all tested concentrations of ZnO NPs from 24 h. The maximum cell death occurred at 96 h with a reported cell death of 44.3 ± 4%, 69.7 ± 2.1%, 83.8 ± 0.9%, 86.7 ± 1.2%, and 87.3 ± 1% at 10, 50, 100, 150, 200 mg/L of ZnO NPs respectively (Fig. 3). The results showed a typical concentration- and time-dependent loss in cell viability upon interaction with ZnO NPs.

The EC_10_ and EC _50_ values for ZnO NPs on the algal growth inhibition are presented in Table 1. The present study reported 1.29 and 31.56 mg/L as the 72 h EC_10_ and EC _50_ values for ZnO NPs on *Spirulina* cells respectively.

**Table 1:** EC_10_ and EC_50_ values of ZnO NPs on S. *platensis* from 48 to 96 h with 95% confidence limits values.

Duration	48 h	72 h	96 h
EC_10_ (mg/L)	3.65 (0.0–16.17)	1.29 (0.29–3.1)	0.83 (0.2–1.98)
EC_50_ (mg/L)	67.87 (13.6–247.3)	31.56 (20.68–43.04)	13.97 (8.02–20.3)

#### Reduction in algal biomass

**Figure 4:**
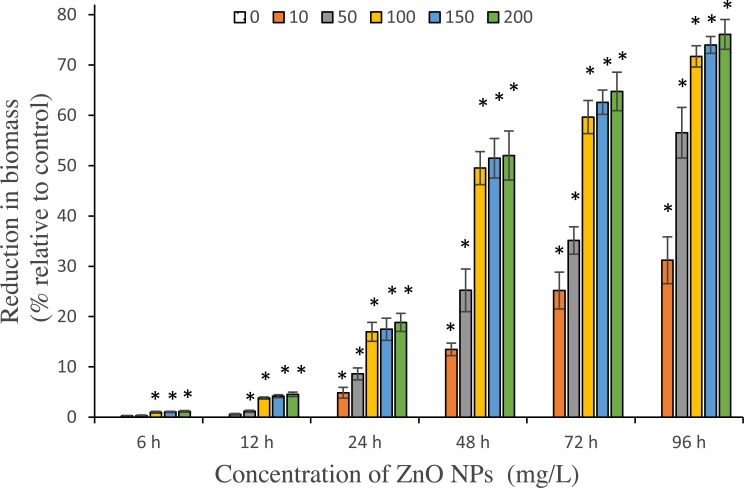
Reduction in biomass. The percentage of reduction in algal biomass by different concentrations of ZnO NPs from 6 to 96 h. *, significant difference at *p* < 0.05 between the control and the tested concentrations at the specific time period.

The reduction in algal biomass due to ZnO NPs treatment exhibited same trend with loss in cell viability (Fig. 4). Results showed a significant (*p* < 0.05%) reduction in algal biomass starting from 24 h among all tested concentrations of ZnO NPs. The algal cells interacted with NPs for 96 h was reported to have maximum reduction in biomass with 31.2 ± 2.7%, 56.5 ± 2.9%, 71.1 ± 1.2%, 74 ± 0.97% and 76.1 ± 1.7% loss in biomass at 10, 50, 100, 150 and 200 mg/L respectively.

#### Decrease in photosynthetic pigments

**Figure 5:**
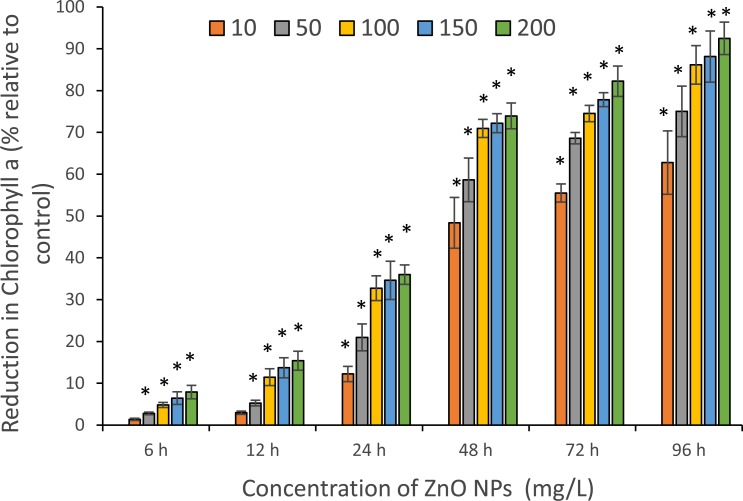
Reduction in chlorophyll a. The percentage of reduction in chlorophyll-a for the different concentrations of ZnO NPs from 6 to 96 h. *, significant difference at *p* < 0.05 between the control and the tested concentrations at the specific time period.

**Figure 6:**
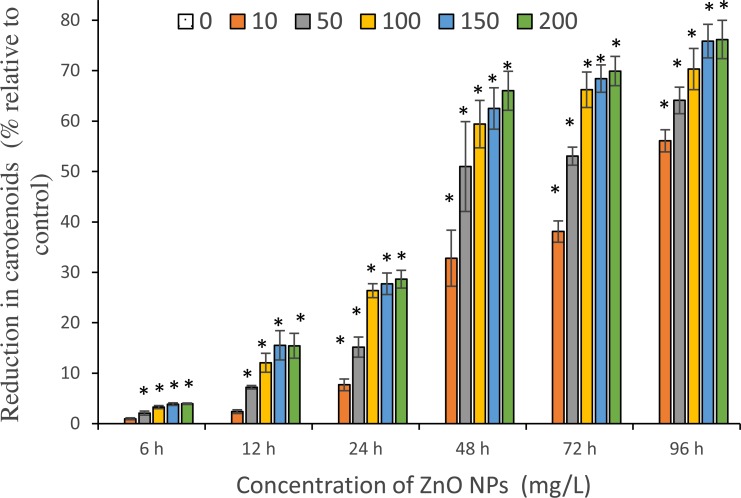
Reduction in carotenoids. The percentage of reduction in carotenoids of *S. platensis* with ZnO NPs treatment from 6 to 96 h. *, significant difference at *p* < 0.05 between the control and the tested concentrations at the specific time period.

The toxicity of ZnO NPs was further confirmed by quantifying the photosynthetic pigments: chlorophyll-a (Chl-a), carotenoids and phycocyanin. The maximum reduction in Chl-a content was reported at 96 h and estimated to be 62.8 ± 4.4%, 75 ± 3.5%, 86.1 ± 2.7%, 88.1 ± 3.5%, and 92.5 ± 2.2% for 10, 50, 100 ,150 and 200 mg/L of ZnO NPs, respectively (Fig. 5). A similar trend was observed with carotenoids that showed a highest fall in carotenoids content at 96 h with the resultant values of 56.1 ± 1.3%, 64.1 ± 1.5%, 70.3 ± 2.4%, 75.9 ± 1.9%, and 76.2 ± 2.2% for 10, 50, 100 ,150 and 200 mg/L of ZnO NPs, respectively (Fig. 6).

**Figure 7:**
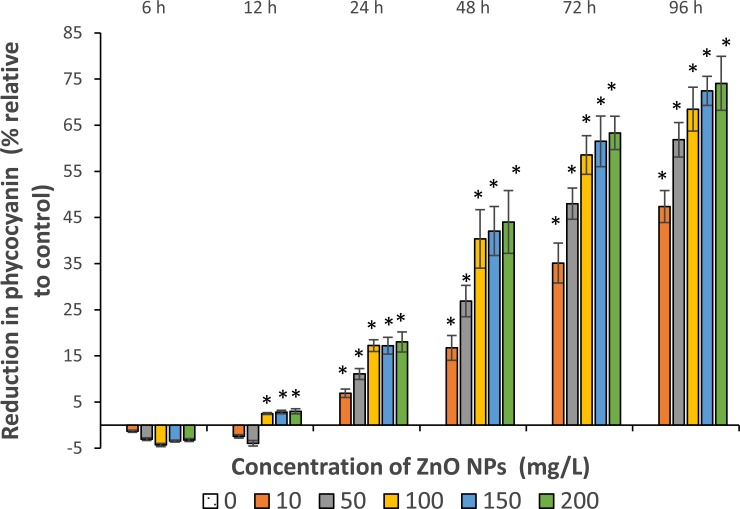
Reduction in phycocyanin. The percentage reduction in phycocyanin pigment of *S. platensis* for the treatment with 10, 50, 100, 150 and 200 mg/L of ZnO NPs for the exposure duration from 6 to 96 h. *, significant difference at *p* < 0.05 between the control and the tested concentrations at the specific time period.

Whereas, the toxic effects of ZnO NPs on the phycocyanin content of cyanobacterial cells showed a slight increase in pigment content for all tested concentrations of ZnO NPs at 6 h and also for 10 and 50 mg/L of ZnO NPs at 12 h. However, reduction in phycocyanin content was observed from 100 to 200 mg/L of ZnO NPs at 12 h. The maximum reduction was noticed at 96 h with the reported values of 47.4 ± 2%, 61.8 ± 2.2%, 68.5± 2.7%, 72.4 ± 1.8%, 74.1 ± 3.4% at 10, 50, 100 ,150 and 200 mg/L of ZnO NPs respectively (Fig. 7).

A significant (*p* < 0.05) reduction in all three photosynthetic pigments was reported among all tested concentrations of ZnO NPs from 24 h onwards. The percentage reduction in photosynthetic pigments for an exposure duration from 6 to 96 h to 10, 50, 100, 150 and 200 mg/L of ZnO NPs showed a sequential increase in pigment loss as the interaction time increased.

### Surface interactions of ZnO NPs on algal cells

**Figure 8:**
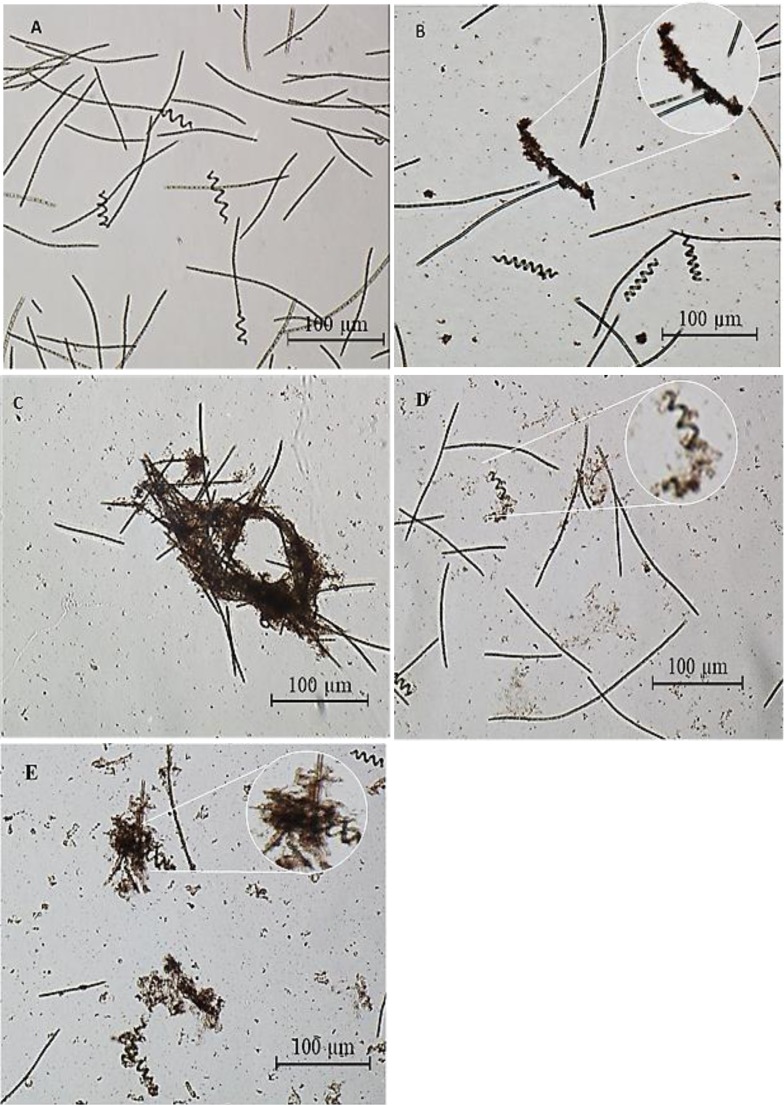
Light microscopic images. Phase contrast microscopic image****(10x) of the control cells without ZnO NPs (A), ZnO NPs (200 mg/L) treated *S. platensis* cells showing encapsulation of algal cells with ZnO NPs agglomerates at 24 h (B), flocculation of algal cells at 48 h (C), fragmentation of algal trichrome at 72 h (D) and distorted cells at 96 h (E).

Figure 8 shows the morphology of the algal cells examined through optical inverted microscope before and after treated with 200 mg/L of ZnO NPs from 24 to 96 h. Figure 8A shows that the control cells were not aggregated with intact cell membrane, whereas ZnO NPs treated cells showed encapsulation of cells by NP agglomerates (Fig. 8B), flocculation of cells (Fig. 8C), fragmentation of trichrome (Fig. 8D) and cell death (Fig. 8E).

**Figure 9:**
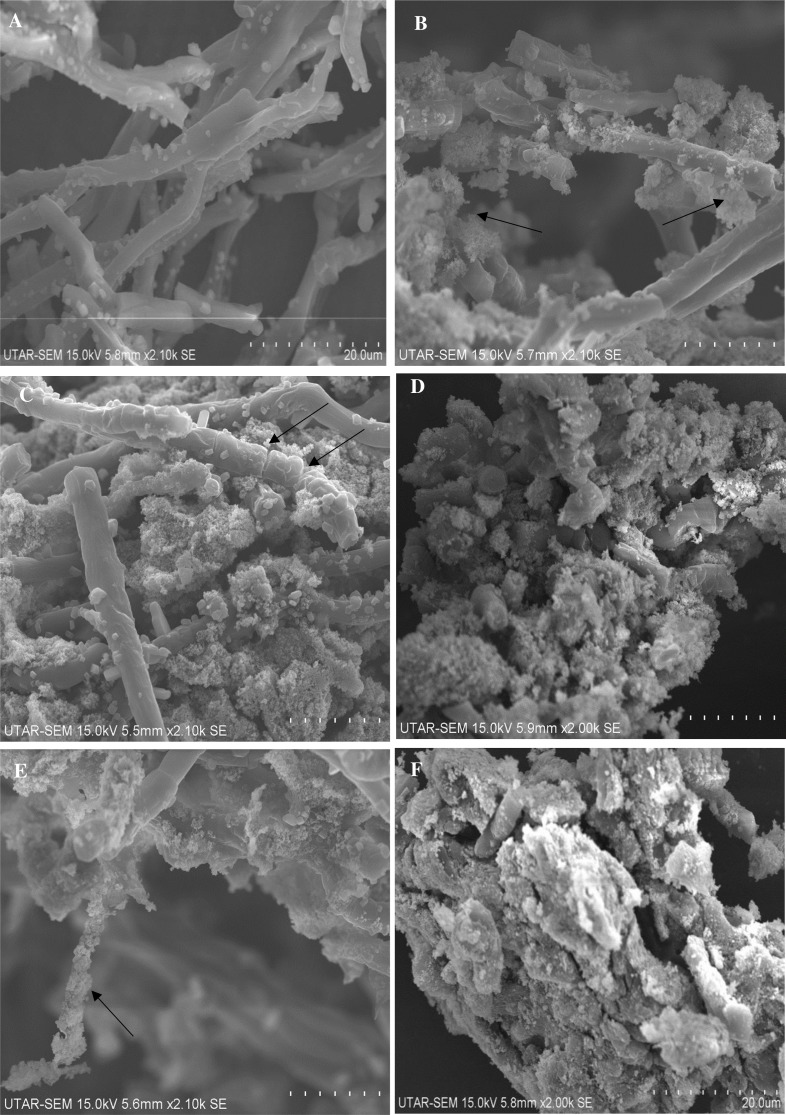
SEM pictures. SEM image of control cells (A) and cells treated with 200 mg/L of ZnO NPs at 96 h showing encapsulation of ZnO NPs agglomerates on algal cells (B), cell membrane rupture and fragmentation of trichrome (C), aggregation of algal cells (D), distorted cell (E) and aggregates of distorted cells (F). Scale bars are in 20 µm.

**Figure 10:**
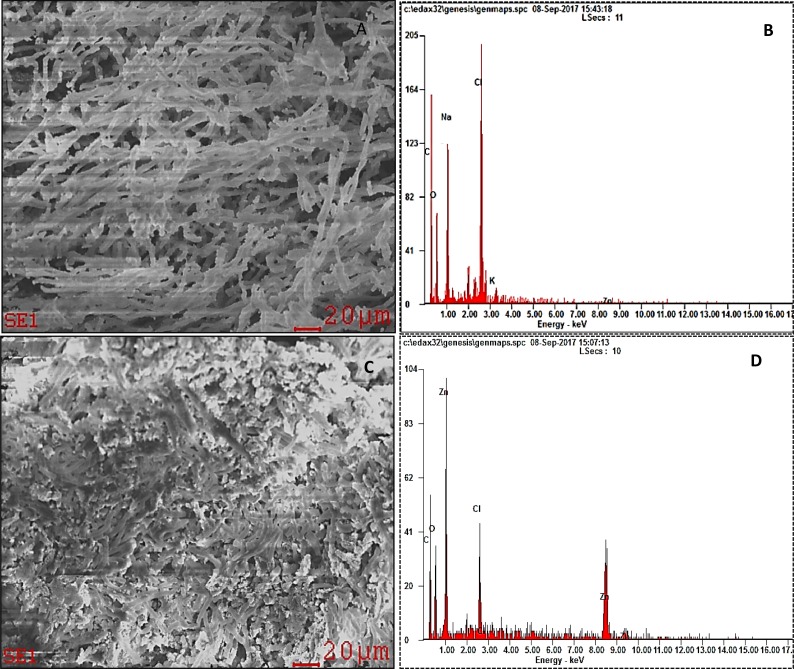
SEM EDX pictures. SEM image obtained from the control algal biomass (A) and EDX spectrum of control sample showing no visible peaks for zinc (B). SEM image of algal biomass treated with 200 mg/L of ZnO NPs for 96 h duration shows the surface accumulation of ZnO NPs in algal biomass (C) and EDX spectrum showing the presence of visible peaks of zinc in the biomass (D).

On the other hand, SEM image of the control cells showed filamentous cells with smooth surface and uncompromised cell membrane (Fig. 9A). In contrast, ZnO NPs treated cells showed the following phenomena such as; adsorption and aggregation of NP agglomerates on algal cells (Fig. 9B), cell wall breakage and fragmentation of trichrome (Fig. 9C), aggregation of cells (Fig. 9D), distorted cells (Fig. 9E) and aggregates of distorted cells (Fig. 9F). Further, SEM EDX spectrum of ZnO NPs interacted cells evidenced aggregation of cells due to the surface accumulation of ZnO NPs on algal cells (Fig. 10C) and the peaks representative for zinc in the EDX spectrum (Fig. 10D) confirmed the presence of Zn in algal cells treated with ZnO NPs.

We noticed that the *S. platensis* used in our study exhibited linear shapes in the culture. Wang & Zhao (2005) reported that the linearization of *Spirulina* filament often occurs in laboratories and mass cultures, and this linear structure of *Spirulina* cells has been now widely accepted as one of the *Spirulina* morphologies. Further, the authors confirmed that the ultramicroscopic study of both the linear and the helical filaments were consistent with the typical characteristic of the original *S. platensis* (Wang & Zhao, 2005).

### Transmission electron microscopic study of ZnO NPs treated algal cells

**Figure 11:**
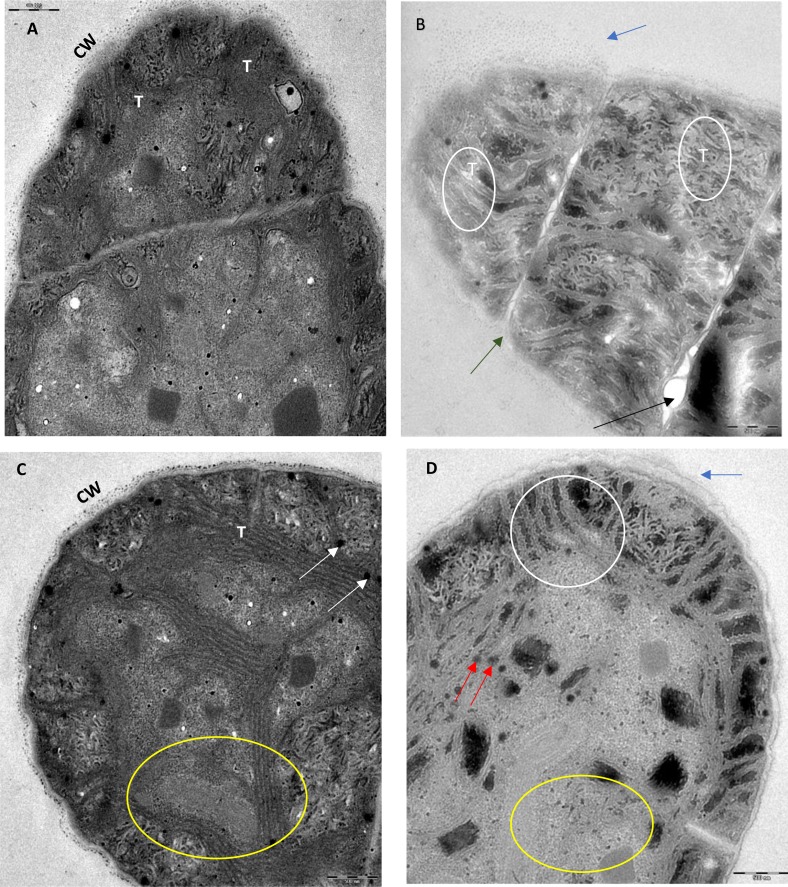
TEM images. Transmission electron micrograph of *S. platensis* cells. The longitudinal (A) and transverse section (C) of the control cells without ZnO NPs treatment displayed the healthy intact cell wall (CW), densely packed thylakoids (T) and the photosynthetic pigment phycobilisomes (white arrowhead). The longitudinal (B) and transverse section (D) of cyanobacterial cells treated with 200 mg/L of ZnO NPs for 96 h displayed the cell wall degradation shown by electron transparent hallow around the cell (blue arrowhead), membrane rupture (green arrowhead), rupture of trichrome (black arrowhead), degradation of thylakoid lamellae (white circle) and degradation of phycobilisomes (red arrowhead). The area under yellow circle in (D) shows the interspersed electron transparent cytoplasm due to the degradation of cytoplasmic organelles compared to the compact electron dense cytoplasm of control cell shown in the area under yellow circle in (C). Scale bars are 500 nm.

TEM micrographs of control cells (Figs. 11A and 11C) showed a dense intact cell wall and large number of concentric thylakoid lamellae, parallel to the long axis of the trichrome, with thylakoids-associated, round-structured phycobilisomes. On the contrary, algal cells treated with 200 mg/L of ZnO NPs for 96 h showed degradation and rupture of cell wall, fragmentation of trichrome, destruction of thylakoids and degradation of photosynthetic pigment phycobilisomes (Figs. 11B and 11D).

### FTIR spectroscopy of *Spirulina* cells interacted with ZnO NPs

**Figure 12:**
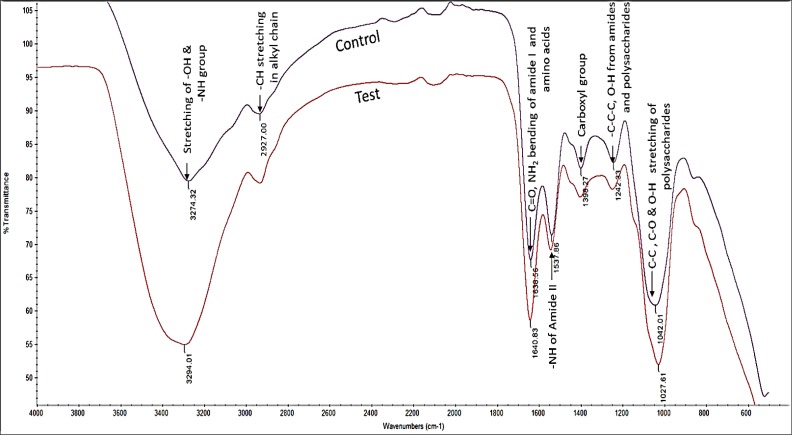
FTIR spectrum. Normalized FTIR spectrum of *Spirulina* cells treated with 200 mg/L of ZnO NPs at 96 h.

The FTIR spectrum obtained from the control algal cells confirmed the presence of numerous functional groups such as amino, carboxyl and hydroxyl groups on the cell surface (Fig. 12), that can mediate the binding of positively charged Zn ions on algal surface (Cardoso et al., 2012). The spectrum from ZnO NPs treated algal cells showed very strong absorption bands at 3,294, 1,640 and 1,027 cm^−1^. The band at 3,294 cm^−1^ may be attributed to N–H stretching coupled with O-H stretching. The peak at 1,640 cm^−1^ corresponded to the coupled vibrations of C = O, and C = N stretching with NH_2_ bending of amide and amino acids, while another peak at 1,027 cm^−1^ suggested the presence of C–O stretching of polysaccharides (Table 2). The presence of strong absorption peaks in ZnO NPs treated algal cells indicated the possible interaction of hydroxyl, amine and carboxyl groups of algal cell surface with ZnO NPs.

**Table 2:** Functional groups involved in the interaction of ZnO NPs on the algal cells.

Peak shift (Wave number cm^−1^)	Bond/Functional group	Component
3,294–3,274	N–H	Amines
2,929–2,927	C–H	Alkyl chain
1,640–1,638	C=O, C–N	Protein primary amides
1,537–1,539	N–H	Secondary amides
1,398–1,400	COO–	Carboxyl group
1,242–1,244	C–O–C, O–H	Amides, Polysaccharides
1,042–1,027	C–C/C–O, O–H	Polysaccharides

## Discussion

From the results of the present study, the potential cytotoxic effects of ZnO NPs on *S. platensis* are quite evident. Our results showed a typical concentration- and time-dependent loss in cell viability and algal biomass due to ZnO NPs treatment (Figs. 3 and 4). We reported higher cytotoxicity with 30.8% and 77.8% of cell death, in comparing to the results of a previous study by Suman, Rajasree & Kirubagaran (2015), where ZnO NPs had caused 19.7% and 64.3% of cell death on freshwater microalga *Chlorella vulgaris* for 24 and 72 h respectively at 200 mg/L. Similarly, our findings showed a higher percentage of algal biomass inhibition of 71.7% on day 4, when compared to the study results of Gunawan et al. (2013), which reported 57.1% inhibition of biomass on green microalga *Chlamydomonas reinhardtii* on day 8 at 100 mg/L of ZnO NPs. A study by Hu et al. (2013) reported 21.1% of growth inhibition on marine alga *Spirodela polyrhiza*., whereas, our study on marine microalga *S. platensis* showed 69.7% of cell death and 56.5% reduction in biomass with 50 mg/L of ZnO NPs at 96 h. These findings indicated the higher sensitivity of *S. platensis* to ZnO NPs toxicity through higher percentage of growth inhibition.

Previous toxicity studies have shown the growth inhibitory effect of heavy metals and metallic nanoparticles on *S. platensis.* For examples, ZnO NPs toxicity had caused 41.0% decrease in biomass yield on day 10 when treated with 10 ppm ZnO NPs (Lone et al., 2013), while the treatment of titanium dioxide NPs resulted in 74.1% reduction in biomass on day 15 at 100 mg/L (Comotto et al., 2014). A treatment with selenium at 100 mg/L was reported to cause 75.6%, 69% and 98.8% reduction in polysaccharides, protein and lipid contents respectively after 72 h of contact that indicated degradation and lysis of algal biomass (Zinicovscaia et al., 2013). Another treatment with 1 mg/L of copper resulted in reduction of 50% biomass on day 7 (Deniz, Saygideger & Karaman, 2011). Based on the above evidences, it can be inferred that heavy metals and metal oxide nanoparticles can exert a major detrimental effect on the growth and nutritional values of *S. platensis*.

The EC_50_ values reported in our study were 31.56 and 13.97 mg/L at 72 and 96 h respectively (Table 1). Whereas, Ji, Long & Lin (2011) reported the EC_30_ value of 20 mg/L for ZnO NPs on microalga *Chlorella* sp., on day 6. This further confirms the higher sensitivity of *S. platensis* than *Chlorella* sp. towards ZnO NPs toxicity. However, the present study exhibited much lower sensitivity of *S. platensis* to ZnO NPs on comparing with the study results of Aruoja et al. (2009) and Franklin et al. (2007) who reported the 72 h EC_50_ value of 0.042 and 0.068 mg/L for nano ZnO to the microalga *Pseudokirchneriella subcapitata* respectively. Similarly, marine microalga *Dunaliella tertiolecta* showed the greater growth inhibition with 96 h EC_50_ value of 2.42 mg/L for ZnO NPs (Ma, Williams & Diamond, 2013; Hou et al., 2018).

The light microscopic and SEM images of ZnO NPs treated cells (Figs. 8 and 9) displayed encapsulation of algal cells with NP agglomerates, damaged cell membrane, fragmentation of trichrome and aggregation of cells with distorted morphology. Similar findings were reported on freshwater microalga *C. vulgaris* (Suman, Rajasree & Kirubagaran, 2015) and *Scenedesmus obliquus* (Bhuvaneshwari et al., 2015) upon treating with ZnO NPs. The formation of large aggregates of NPs treated cells was possibly due to the self -protecting mechanism of algal cells such as secretion of exudates (exopolysaccharides) from stressed cells which ultimately caused flocculation of cells (Sadiq et al., 2011) and reduction in surface area of algal cells to prevent binding of metal ions. The adsorption of ZnO NPs on algal cells was possibly due to interactions with the algal surface biomolecules such as proteins, polysaccharides and lipids (Chen et al., 2012). The adsorption and aggregation of ZnO NPs on algal cells compromised the cell morphology, membrane integrity and thus the cell viability due to mechanical damage or by release of zinc ions that disturbs the cellular metabolism of algal cells (Hazeem et al., 2016; Lin & Xing, 2008).

Furthermore, the study results showed the decrease in the photosynthetic pigments, Chl-a, carotenoids and phycocyanin, of *S. platensis* corresponding to algal growth inhibition caused by ZnO NPs toxicity. We reported a characteristic concentration and time-dependent decrease in photosynthetic pigments. A slight increase in phycocyanin content was observed during the early hours (6–12 h) of treatment with ZnO NPs that may be due to the acute stress triggered by the nanoparticles on algal cells. However, a significant reduction in Chl-a, carotenoids and phycocyanin contents was reported from 24 h onwards among all tested concentrations of ZnO NPs. The highest percentage of reduction in Chl-a followed by carotenoids and phycocyanin was reported at 96 h with 200 mg/L with the resultant values of 92.5%, 76.2% and 74.1% respectively (Figs. 5–7). Our findings were similar to previous studies on *S. platensis* which reported 93.5% and 50% reduction in Chl-a and carotenoids respectively on day 10 with 10 mg/L of ZnO NPs (Lone et al., 2013), and 53.1%, 37.5% and 32.1% reduction in Chl-a, carotenoids and phycocyanin by the salt stress of sea water on day 25 (Leema et al., 2010). A recent study by Zinicovscaia et al. (2016) reported 90% loss in phycocyanin pigment of *S. platensis* after 72 h of contact time with 100 mg/L of selenium ions.

Moreover, TEM micrographs (Fig. 11) of ZnO NPs treated algal cells confirmed the damage to the photosynthetic system through remarkable reduction and degradation of thylakoid lamellae and photosynthetic pigment phycobilisomes. Similar findings were reported in TEM micrography of *S. platensis* cells treated with lysozyme enzyme (Vladimirescu, 2010) and the UV irradiated cyanobacterium *Cylindrospermopsis raciborskii* (Noyma et al., 2015) that showed cell wall degradation and thylakoids damage. The destruction of photosynthetic system is believed to be the major adverse effect of ZnO NPs on algal cells which usually occurs as a result of oxidative stress (Deniz, Saygideger & Karaman, 2011). Also, loss in photosynthetic pigments results in reduction of the photosynthetic electron flow that leads to cell death due to failure of the photosynthesis process (Mohanty, 1989).

In addition to destruction of the photosynthetic apparatus, TEM images showed that the interaction of ZnO NPs on algal cells resulted in cell wall damage and degradation of cytoplasmic organelles. Our results are in accordance with the previous findings by Bhuvaneshwari et al. (2015) and Dalai et al. (2014) who treated *Scenedesmus obliquus* with ZnO NPs and titanium dioxide NPs respectively.

The interaction of ZnO NPs on the algal cells was further confirmed by the FTIR spectrum obtained from ZnO NPs treated cells. Our results demonstrated the possible participation of hydroxyl, carboxy and amino groups of polysaccharides and proteins of algal cell wall in binding of ZnO NPs on the cells (Fig. 12 and Table 2). This may be due to binding of positively charged zinc ions to the amino and anionic groups of algal cell wall through electrostatic attraction. Our findings were similar with the earlier reports on *S. platensis* when treated with copper and cadmium (Celekli, Yavuzatmaca & Bozkurt, 2010; Fang et al., 2011).

Researchers have proposed the following reasons for growth inhibition of the algal cells: Shading effect and adsorption of NPs on the cellular surface decrease the light intensity reaching the cells, thereby inhibiting the photosynthetic activity (Navarro et al., 2008; Van Hoecke et al., 2008), blockage of nutrient intake by densely packed NPs on the algal surface hinders its photosynthetic efficiency (Lin et al., 2009), penetration of NPs into cell envelope disrupts the cell membrane, which attributes to cell growth inhibition (Brayner et al., 2006), and the toxicity of ZnO NPs to the algal cells is solely due to the dissolved Zn^+^ from ZnO NPs and this causes the higher toxicity of ZnO NPs compared to other metal oxide NPs (Ma, Williams & Diamond, 2013).

Overall, the present study exhibited a typical concentration- and time-dependent cytotoxicity of ZnO NPS on cell viability, algal biomass and the photosynthetic pigments of *S. platensis* with a significant (*p* < 0.05) reduction in all toxicity parameters for 10–200 mg/L of ZnO NPs from 24 h onwards. Our findings were in accordance with the study results of Choudhary et al. (2007), Sadiq et al. (2011) and Suman, Rajasree & Kirubagaran (2015), where the treatement of ZnO NPs showed time- and dose-dependent growth inhibition of *S. platensis* by Zn ions, and *C. vulgaris* by aluminum oxide NPs and ZnO NPs respectively. Besides, our results showed a substantial reduction in all studied cytotoxicity parameters at an exposure time of 48 h. A similar finding was reported in reduction of phycocyanin from *S. platensis* by selenium ions (Zinicovscaia et al., 2016), this may be due to highest dissolution of zinc ions from ZnO NPs at 48 h which was recently reported by Bhuvaneshwari et al. (2015).

## Conclusion

In summary, the treatment of nano scale ZnO particles on *S. platensis* exhibited great effect on their toxicity level, which increased as the concentration of ZnO NPs and time of exposure increased. The study reported a statistically significant reduction in cell viability, algal biomass and the photosynthetic pigments at 10, 50, 100, 150 and 200 mg/L of ZnO NPs from 24 h onwards. The reduction in photosynthetic pigments and cell growth might be due to oxidative stress induced by the interaction of ZnO NPs on algal cells, which led to cell death. The findings from this study recommend the cyanobcterium *S. platensis* to function as an organism model for ecotoxicity assessment, particularly on ZnO NPs in aquatic environments including marine ecosystems, as early as 24 h of contact time.

##  Supplemental Information

10.7717/peerj.4682/supp-1 Data S1 Raw data for cell death

10.7717/peerj.4682/supp-2 Data S2 Raw data for algal biomass reduction

10.7717/peerj.4682/supp-3 Data S3 Raw data for chlorophyll a reduction

10.7717/peerj.4682/supp-4 Data S4 Raw data for carotenoids reduction

10.7717/peerj.4682/supp-5 Data S5 Raw data for phycocyanin reduction

## Additional Information and Declarations

### Data Availability

The following information was supplied regarding data availability: The raw data are provided in the Supplemental Files.
