# Supplementary material for: Cytotoxic effects of zinc oxide nanoparticles on cyanobacterium Spirulina (Arthrospira) platensis
Source: PeerJ. 2018 Jun 1;6:e4682. doi: 10.7717/peerj.4682 (PMC5985776; doi:10.7717/peerj.4682)
Supplement: Data S1 [file peerj-06-4682-s001.docx]

| Cell death (%) | | | | | | |
| --- | --- | --- | --- | --- | --- | --- |
| Conc. of ZnO NPs (mg/L) |  | 10 | 50 | 100 | 150 | 200 |
| Duration of treatment |  |  |  |  |  |  |
| 6 h |  |  |  |  |  |  |
|  | Replicate 1 | 0.454545 | 0.454545 | 2.207792 | 2.792208 | 2.597403 |
|  | Replicate 2 | 0.162338 | 0.649351 | 1.623377 | 2.597403 | 3.571429 |
|  | Replicate 3 | 0.25974 | 0.551948 | 1.233766 | 1.623377 | 2.792208 |
|  | Mean | 0.292208 | 0.551948 | 1.688312 | 2.337662 | 2.987013 |
|  | Std. Devi | 0.148785 | 0.097403 | 0.490249 | 0.626211 | 0.515406 |
|  | Std. Error | 0.085901 | 0.056235 | 0.283045 | 0.361543 | 0.29757 |
| 12 h | 12 h |  |  |  |  |  |
|  | Replicate 1 | 1.987578 | 4.037267 | 7.763975 | 8.695652 | 9.813665 |
|  | Replicate 2 | 1.614907 | 3.10559 | 8.695652 | 7.763975 | 10.55901 |
|  | Replicate 3 | 1.055901 | 2.732919 | 6.832298 | 9.161491 | 9.440994 |
|  | Mean | 1.552795 | 3.291925 | 7.763975 | 8.540373 | 9.937888 |
|  | Std. Devi | 0.468934 | 0.671842 | 0.931677 | 0.71158 | 0.569264 |
|  | Std.Error | 0.270739 | 0.387888 | 0.537904 | 0.410831 | 0.328665 |
| 24 h |  |  |  |  |  |  |
|  | Replicate 1 | 10 | 21.66667 | 32.5 | 27.5 | 35.83333 |
|  | Replicate 2 | 8 | 16.33333 | 26.66667 | 31.66667 | 26.66667 |
|  | Replicate 3 | 9.166667 | 17.83333 | 23.33333 | 25 | 30 |
|  | Mean | 9.055556 | 18.61111 | 27.5 | 28.05556 | 30.83333 |
|  | Std. Devi | 1.004619 | 2.750421 | 4.639804 | 3.367877 | 4.639804 |
|  | Std.Error | 0.580017 | 1.587956 | 2.678792 | 1.944444 | 2.678792 |
| 48 h |  |  |  |  |  |  |
|  | Replicate 1 | 22.8972 | 41.1215 | 63.5514 | 69.85981 | 71.96262 |
|  | Replicate 2 | 21.35514 | 32.71028 | 66.35514 | 66.35514 | 63.20093 |
|  | Replicate 3 | 27.80374 | 36.91589 | 62.85047 | 64.25234 | 65.65421 |
|  | Mean | 24.01869 | 36.91589 | 64.25234 | 66.82243 | 66.93925 |
|  | Std. Devi | 3.367405 | 4.205607 | 1.854499 | 2.832793 | 4.519986 |
|  | Std.Error | 1.944172 | 2.428109 | 1.070695 | 1.635514 | 2.609615 |
| 72 h |  |  |  |  |  |  |
|  | Replicate 1 | 34.78261 | 53.84615 | 66.88963 | 75.41806 | 80.43478 |
|  | Replicate 2 | 32.27425 | 43.2107 | 70.90301 | 79.43144 | 77.67559 |
|  | Replicate 3 | 39.29766 | 47.82609 | 74.91639 | 72.65886 | 75.41806 |
|  | Mean | 35.45151 | 48.29431 | 70.90301 | 75.83612 | 77.84281 |
|  | Std. Devi | 3.559163 | 5.333164 | 4.013378 | 3.405587 | 2.512538 |
|  | Std.Error | 2.054884 | 3.079103 | 2.317125 | 1.966217 | 1.450615 |
| 96 h |  |  |  |  |  |  |
|  | Replicate 1 | 50.686 | 73.59565 | 84.85633 | 88.73932 | 89.20528 |
|  | Replicate 2 | 45.24981 | 66.45094 | 84.54569 | 84.58452 | 86.87549 |
|  | Replicate 3 | 37.01786 | 69.13021 | 82.02174 | 86.79783 | 85.71059 |
|  | Mean | 44.31789 | 69.7256 | 83.80792 | 86.70722 | 87.26378 |
|  | Std. Devi | 6.881557 | 3.609373 | 1.554652 | 2.078882 | 1.779411 |
|  | Std.Error | 3.973069 | 2.083873 | 0.897579 | 1.200243 | 1.027343 |
