# Supplementary material for: Cytotoxic effects of zinc oxide nanoparticles on cyanobacterium Spirulina (Arthrospira) platensis
Source: PeerJ. 2018 Jun 1;6:e4682. doi: 10.7717/peerj.4682 (PMC5985776; doi:10.7717/peerj.4682)
Supplement: Data S2 [file peerj-06-4682-s002.docx]

| Reduction in biomass (%) | | | | | | |
| --- | --- | --- | --- | --- | --- | --- |
| Conc. of ZnO NPs | | 10 | 50 | 100 | 150 | 200 |
| Duration of treatment |  |  |  |  |  |  |
| 6 h | Replicate 1 | 0.250752 | 0.341023 | 0.792377 | 0.942828 | 1.12337 |
|  | Replicate 2 | 0.341023 | 0.401204 | 1.15346 | 1.003009 | 1.003009 |
|  | Replicate 3 | 0.160481 | 0.100301 | 1.003009 | 1.183551 | 1.334002 |
|  | Mean | 0.250752 | 0.280843 | 0.982949 | 1.043129 | 1.15346 |
|  | Std. Devi | 0.090271 | 0.159223 | 0.181376 | 0.125276 | 0.167536 |
|  | Std. Error | 0.052118 | 0.091927 | 0.104717 | 0.072328 | 0.096727 |
| 12 h |  |  |  |  |  |  |
|  | Replicate 1 | 0.655405 | 1.196911 | 3.571429 | 4.440154 | 4.440154 |
|  | Replicate 2 | 0.3861 | 0.965251 | 3.861004 | 3.861004 | 5.019305 |
|  | Replicate 3 | 0.675676 | 1.370656 | 3.976834 | 4.208494 | 4.150579 |
|  | Mean | 0.572394 | 1.177606 | 3.803089 | 4.169884 | 4.53668 |
|  | Std. Devi | 0.161653 | 0.203391 | 0.208816 | 0.291499 | 0.442334 |
|  | Std. Error | 0.09333 | 0.117428 | 0.12056 | 0.168297 | 0.255381 |
| 24 h |  |  |  |  |  |  |
|  | Replicate 1 | 7.455013 | 9.511568 | 18.41432 | 19.43734 | 17.13555 |
|  | Replicate 2 | 5.398458 | 8.74036 | 14.83376 | 15.08951 | 20.71611 |
|  | Replicate 3 | 6.940874 | 11.05398 | 17.64706 | 17.90281 | 18.67008 |
|  | Mean | 6.598115 | 9.768638 | 16.96505 | 17.47656 | 18.84058 |
|  | Std. Devi | 1.070265 | 1.17804 | 1.885196 | 2.205033 | 1.79636 |
|  | Std. Error | 0.617918 | 0.680142 | 1.088418 | 1.273076 | 1.037129 |
| 48 h |  |  |  |  |  |  |
|  | Replicate 1 | 13.75 | 25 | 50.43478 | 49.61957 | 48.6413 |
|  | Replicate 2 | 12.11957 | 21.08696 | 45.86957 | 55.97826 | 57.6087 |
|  | Replicate 3 | 14.56522 | 29.56522 | 52.22826 | 48.80435 | 49.78261 |
|  | Mean | 13.47826 | 25.21739 | 49.51087 | 51.46739 | 52.01087 |
|  | Std. Devi | 1.245265 | 4.243309 | 3.278485 | 3.927735 | 4.88133 |
|  | Std. Error | 0.718954 | 2.449876 | 1.892834 | 2.267679 | 2.818237 |
| 72 h |  |  |  |  |  |  |
|  | Replicate 1 | 21.45868 | 37.77403 | 56.49241 | 64.8398 | 68.25464 |
|  | Replicate 2 | 25.25295 | 32.33558 | 63.06914 | 62.94266 | 60.6661 |
|  | Replicate 3 | 28.79427 | 35.24452 | 59.40135 | 60.03373 | 65.3457 |
|  | Mean | 25.16863 | 35.11804 | 59.6543 | 62.6054 | 64.75548 |
|  | Std. Devi | 3.668518 | 2.721429 | 3.295653 | 2.420721 | 3.828541 |
|  | Std. Error | 2.11802 | 1.571218 | 1.902746 | 1.397604 | 2.210409 |
| 96 h |  |  |  |  |  |  |
|  | Replicate 1 | 30.80495 | 62.0356 | 73.99381 | 75.85139 | 73.87771 |
|  | Replicate 2 | 26.74149 | 55.41796 | 71.32353 | 73.52941 | 74.9226 |
|  | Replicate 3 | 36.02941 | 52.16718 | 69.81424 | 72.60062 | 79.45046 |
|  | Mean | 31.19195 | 56.54025 | 71.71053 | 73.99381 | 76.08359 |
|  | Std. Devi | 4.656041 | 5.029024 | 2.116487 | 1.674405 | 2.962233 |
|  | Std. Error | 2.688166 | 2.903509 | 1.221955 | 0.966718 | 1.710246 |
|  |  |  |  |  |  |  |
