# Supplementary material for: Cytotoxic effects of zinc oxide nanoparticles on cyanobacterium Spirulina (Arthrospira) platensis
Source: PeerJ. 2018 Jun 1;6:e4682. doi: 10.7717/peerj.4682 (PMC5985776; doi:10.7717/peerj.4682)
Supplement: Data S3 [file peerj-06-4682-s003.docx]

| Reduction in chlorophyll a (%) | | | | | | |
| --- | --- | --- | --- | --- | --- | --- |
| Conc. of ZnO NPs | | 10 | 50 | 100 | 150 | 200 |
| Duration of treatment | | |  |  |  |  |
| 6 h |  |  |  |  |  |  |
|  | Replicate 1 | 1.14401 | 2.72205 | 5.313838 | 8.149609 | 6.282548 |
|  | Replicate 2 | 1.306179 | 3.129911 | 4.131012 | 5.27908 | 9.504606 |
|  | Replicate 3 | 1.67071 | 2.539238 | 4.98531 | 5.922373 | 7.885115 |
|  | Mean | 1.373633 | 2.797067 | 4.810053 | 6.450354 | 7.890756 |
|  | Std. Devi | 0.269751 | 0.302398 | 0.610578 | 1.506339 | 1.611036 |
|  | Std. Error | 0.155741 | 0.174589 | 0.352517 | 0.869685 | 0.930132 |
| 12 h |  |  |  |  |  |  |
|  | Replicate 1 | 3.329922 | 5.523704 | 9.630396 | 12.40567 | 13.923 |
|  | Replicate 2 | 2.960501 | 5.790888 | 11.15552 | 16.47973 | 14.24783 |
|  | Replicate 3 | 2.558399 | 4.513216 | 13.61831 | 12.23741 | 18.00386 |
|  | Mean | 2.949607 | 5.275936 | 11.46807 | 13.7076 | 15.39157 |
|  | Std. Devi | 0.385877 | 0.673909 | 2.012244 | 2.402204 | 2.268134 |
|  | Std. Error | 0.222786 | 0.389082 | 1.16177 | 1.386913 | 1.309508 |
|  |  |  |  |  |  |  |
| 24 h | Replicate 1 | 10.19551 | 21.8085 | 31.8141 | 32.90373 | 33.34686 |
|  | Replicate 2 | 12.7529 | 17.43358 | 30.36458 | 31.14341 | 37.72975 |
|  | Replicate 3 | 13.71579 | 23.68463 | 36.06348 | 39.78915 | 36.87258 |
|  | Mean | 12.2214 | 20.97557 | 32.74739 | 34.6121 | 35.98306 |
|  | Std. Devi | 1.819329 | 3.207683 | 2.96186 | 4.569034 | 2.3229 |
|  | Std. Error | 1.05039 | 1.851957 | 1.710031 | 2.637933 | 1.341127 |
| 48 h |  |  |  |  |  |  |
|  | Replicate 1 | 54.33449 | 63.22046 | 68.70323 | 74.81666 | 74.75901 |
|  | Replicate 2 | 48.58405 | 59.74777 | 71.12269 | 71.02608 | 76.54566 |
|  | Replicate 3 | 42.19052 | 52.95743 | 73.03343 | 70.76355 | 70.53175 |
|  | Mean | 48.36968 | 58.64189 | 70.95312 | 72.2021 | 73.94548 |
|  | Std. Devi | 6.074823 | 5.22012 | 2.170074 | 2.26808 | 3.08839 |
|  | Std. Error | 3.507301 | 3.013838 | 1.252893 | 1.309477 | 1.783083 |
| 72 h |  |  |  |  |  |  |
|  | Replicate 1 | 56.53993 | 67.05806 | 76.65373 | 78.85965 | 78.2888 |
|  | Replicate 2 | 56.97712 | 69.23687 | 72.839 | 78.72428 | 82.99333 |
|  | Replicate 3 | 53.019 | 69.55915 | 74.05171 | 75.90802 | 85.43975 |
|  | Mean | 55.51202 | 68.61802 | 74.51481 | 77.83065 | 82.24063 |
|  | Std. Devi | 2.170054 | 1.360547 | 1.949073 | 1.666423 | 3.634415 |
|  | Std. Error | 1.252881 | 0.785512 | 1.125298 | 0.96211 | 2.09833 |
| 96 h |  |  |  |  |  |  |
|  | Replicate 1 | 71.25404 | 81.72939 | 88.09098 | 92.80384 | 95.13279 |
|  | Replicate 2 | 60.47822 | 73.34479 | 80.88757 | 90.39339 | 94.30957 |
|  | Replicate 3 | 56.61874 | 70.00109 | 89.46101 | 81.2078 | 88.0478 |
|  | Mean | 62.78367 | 75.02509 | 86.14652 | 88.13501 | 92.49672 |
|  | Std. Devi | 7.585139 | 6.042004 | 4.605612 | 6.119007 | 3.874803 |
|  | Std. Error | 4.379282 | 3.488352 | 2.659052 | 3.53281 | 2.237119 |
|  |  |  |  |  |  |  |
