# Supplementary material for: Cytotoxic effects of zinc oxide nanoparticles on cyanobacterium Spirulina (Arthrospira) platensis
Source: PeerJ. 2018 Jun 1;6:e4682. doi: 10.7717/peerj.4682 (PMC5985776; doi:10.7717/peerj.4682)
Supplement: Data S4 [file peerj-06-4682-s004.docx]

| Reduction in carotenoids (%) | | | | | | |
| --- | --- | --- | --- | --- | --- | --- |
| Conc. of ZnO NPs (mg/L) | 6 h | 10 | 50 | 100 | 150 | 200 |
| Duration of treatment |  |  |  |  |  |  |
| 6 h |  |  |  |  |  |  |
|  | Replicate 1 | 0.72096 | 1.758798 | 3.402829 | 4.140369 | 3.810369 |
|  | Replicate 2 | 0.578919 | 2.5291 | 2.92805 | 3.833462 | 4.103462 |
|  | Replicate 3 | 0.345394 | 1.964003 | 3.500185 | 3.62051 | 3.92051 |
|  | Mean | 0.548424 | 2.083967 | 3.277021 | 3.86478 | 3.94478 |
|  | Std. Devi | 0.189631 | 0.398917 | 0.306113 | 0.261341 | 0.148046 |
|  | Std.Error | 0.109484 | 0.230315 | 0.176735 | 0.150885 | 0.085475 |
| 12 h |  |  |  |  |  |  |
|  | Replicate 1 | 2.79139 | 7.576013 | 10.84295 | 16.05943 | 13.06846 |
|  | Replicate 2 | 2.263026 | 7.161499 | 11.12879 | 18.14386 | 17.99908 |
|  | Replicate 3 | 2.076671 | 6.895444 | 14.23896 | 12.40119 | 15.24384 |
|  | Mean | 2.377029 | 7.210986 | 12.07023 | 15.53483 | 15.43713 |
|  | Std. Devi | 0.370747 | 0.342973 | 1.883601 | 2.907052 | 2.470984 |
|  | Std .Error | 0.214051 | 0.198015 | 1.087497 | 1.678387 | 1.426623 |
| 24 h |  |  |  |  |  |  |
|  | Replicate 1 | 7.406746 | 17.41403 | 26.53568 | 25.66689 | 28.21322 |
|  | Replicate 2 | 6.702237 | 13.59664 | 24.90322 | 29.9181 | 27.14049 |
|  | Replicate 3 | 8.969711 | 14.50597 | 27.65375 | 27.62841 | 30.57549 |
|  | Mean | 7.692898 | 15.17222 | 26.36421 | 27.7378 | 28.64307 |
|  | Std. Devi | 1.160505 | 1.993998 | 1.383257 | 2.127716 | 1.75738 |
|  | Std .Error | 0.670018 | 1.151235 | 0.798624 | 1.228437 | 1.014624 |
| 48 h |  |  |  |  |  |  |
|  | Replicate 1 | 38.24832 | 40.70508 | 55.50312 | 61.76396 | 61.82343 |
|  | Replicate 2 | 27.12914 | 56.48817 | 58.07317 | 58.80802 | 66.74343 |
|  | Replicate 3 | 33.02692 | 55.76239 | 64.62253 | 66.91385 | 69.46238 |
|  | Mean | 32.80146 | 50.98522 | 59.39961 | 62.49528 | 66.00975 |
|  | Std. Devi | 5.563017 | 8.91025 | 4.702181 | 4.102101 | 3.871964 |
|  | Std .Error | 3.211809 | 5.144335 | 2.714805 | 2.368349 | 2.235479 |
| 72 h |  |  |  |  |  |  |
|  | Replicate 1 | 39.96809 | 54.59653 | 62.48557 | 66.44529 | 72.15371 |
|  | Replicate 2 | 38.5053 | 53.46172 | 66.69292 | 67.32053 | 66.64543 |
|  | Replicate 3 | 35.79981 | 51.08105 | 69.46332 | 71.52625 | 70.97907 |
|  | Mean | 38.09107 | 53.04644 | 66.21394 | 68.43069 | 69.92607 |
|  | Std. Devi | 2.114792 | 1.794156 | 3.51345 | 2.716317 | 2.901188 |
|  | Std .Error | 1.220976 | 1.035856 | 2.028491 | 1.568266 | 1.675002 |
| 96 h |  |  |  |  |  |  |
|  | Replicate 1 | 58.55893 | 67.09688 | 74.41062 | 79.64019 | 72.82362 |
|  | Replicate 2 | 54.37673 | 62.19104 | 66.24701 | 74.56044 | 75.41909 |
|  | Replicate 3 | 55.30887 | 63.00034 | 70.29644 | 73.36801 | 80.31567 |
|  | Mean | 56.08151 | 64.09609 | 70.31802 | 75.85622 | 76.18613 |
|  | Std. Devi | 2.195548 | 2.63008 | 4.081846 | 3.330815 | 3.804465 |
|  | Std .Error | 1.2676 | 1.518478 | 2.356655 | 1.923047 | 2.196509 |
|  |  |  |  |  |  |  |
