# Supplementary material for: Cytotoxic effects of zinc oxide nanoparticles on cyanobacterium Spirulina (Arthrospira) platensis
Source: PeerJ. 2018 Jun 1;6:e4682. doi: 10.7717/peerj.4682 (PMC5985776; doi:10.7717/peerj.4682)
Supplement: Data S5 [file peerj-06-4682-s005.docx]

| Reduction in phycocyanin (%) | | | | | | |
| --- | --- | --- | --- | --- | --- | --- |
| Conc. of ZnO NPs (mg/L) | | 10 | 50 | 100 | 150 | 200 |
| Duration of treatment | |  |  |  |  |  |
| 6 h |  |  |  |  |  |  |
|  | Replicate 1 | -1.09399 | -2.96723 | -4.20702 | -3.50685 | -3.5701 |
|  | Replicate 2 | -1.55251 | -3.34974 | -4.6957 | -3.63034 | -3.18023 |
|  | Replicate 3 | -1.3356 | -2.76362 | -4.00688 | -3.20885 | -2.93908 |
|  | Mean | -1.32736 | -3.02687 | -4.3032 | -3.44868 | -3.2298 |
|  | Std. Devi | 0.229373 | 0.297578 | 0.354335 | 0.216682 | 0.318418 |
|  | Std. Error | 0.132428 | 0.171807 | 0.204576 | 0.125102 | 0.183839 |
| 12 h |  |  |  |  |  |  |
|  | Replicate 1 | -2.77261 | -4.61256 | 2.717191 | 3.256338 | 2.65067 |
|  | Replicate 2 | -2.05522 | -3.62713 | 2.32252 | 2.502447 | 3.63609 |
|  | Replicate 3 | -2.48038 | -3.52562 | 2.489388 | 2.753596 | 2.734862 |
|  | Mean | -2.43607 | -3.92177 | 2.5097 | 2.83746 | 3.007207 |
|  | Std. Devi | 0.360745 | 0.600388 | 0.198118 | 0.383879 | 0.546253 |
|  | Std. Error | 0.208276 | 0.346634 | 0.114384 | 0.221633 | 0.315379 |
| 24 h |  |  |  |  |  |  |
|  | Replicate 1 | 6.821118 | 10.95425 | 18.55348 | 17.5641 | 15.53842 |
|  | Replicate 2 | 6.03286 | 12.32092 | 17.12657 | 15.24306 | 18.94118 |
|  | Replicate 3 | 7.849562 | 9.980823 | 16.02339 | 18.83869 | 19.5832 |
|  | Mean | 6.90118 | 11.08533 | 17.23448 | 17.21529 | 18.02093 |
|  | Std. Devi | 0.910993 | 1.175541 | 1.268493 | 1.82302 | 2.173753 |
|  | Std. Error | 0.525962 | 0.678699 | 0.732365 | 1.052521 | 1.255017 |
| 48 h |  |  |  |  |  |  |
|  | Replicate 1 | 16.41092 | 24.7443 | 33.38587 | 40.47806 | 46.41494 |
|  | Replicate 2 | 14.22884 | 25.11968 | 45.76623 | 48.00228 | 49.34136 |
|  | Replicate 3 | 19.56894 | 30.81291 | 41.93566 | 37.75481 | 36.3589 |
|  | Mean | 16.73623 | 26.8923 | 40.36258 | 42.07838 | 44.0384 |
|  | Std. Devi | 2.684871 | 3.400535 | 6.338314 | 5.307865 | 6.809701 |
|  | Std. Error | 1.550111 | 1.9633 | 3.659428 | 3.064497 | 3.931583 |
| 72 h |  |  |  |  |  |  |
|  | Replicate 1 | 39.80206 | 49.51831 | 54.53042 | 61.54413 | 59.59696 |
|  | Replicate 2 | 34.34332 | 44.12588 | 62.89135 | 66.9769 | 66.81191 |
|  | Replicate 3 | 31.25299 | 50.36611 | 58.2453 | 56.00776 | 63.6113 |
|  | Mean | 35.13279 | 48.00343 | 58.55569 | 61.5096 | 63.34006 |
|  | Std. Devi | 4.328868 | 3.384705 | 4.189096 | 5.484648 | 3.615113 |
|  | Std. Error | 2.499273 | 1.954161 | 2.418576 | 3.166563 | 2.087186 |
| 96 h |  |  |  |  |  |  |
|  | Replicate 1 | 43.90414 | 58.47778 | 64.76629 | 71.91521 | 67.76869 |
|  | Replicate 2 | 47.3699 | 61.19317 | 66.87551 | 69.58858 | 75.18976 |
|  | Replicate 3 | 50.83841 | 65.8699 | 73.84662 | 75.83186 | 79.31569 |
|  | Mean | 47.37082 | 61.84695 | 68.49614 | 72.44522 | 74.09138 |
|  | Std. Devi | 3.467137 | 3.739174 | 4.752146 | 3.155204 | 5.851334 |
|  | Std. Error | 2.001752 | 2.158813 | 2.743653 | 1.821658 | 3.378269 |
|  |  |  |  |  |  |  |
